# Characterization and evaluation of the enzymatic activity of tetanus toxin submitted to cobalt-60 gamma radiation

**DOI:** 10.1590/1678-9199-JVATITD-2020-0140

**Published:** 2021-04-30

**Authors:** Giselle Pacifico Sartori, Andréa da Costa, Fernanda Lúcio dos Santos Macarini, Douglas Oscar Ceolin Mariano, Daniel Carvalho Pimenta, Patrick Jack Spencer, Luiz Henrique da Silva Nali, Andrés Jimenez Galisteo

**Affiliations:** 1 Laboratory of Protozoology, Institute of Tropical Medicine, University of São Paulo (IMT/FMUSP), São Paulo, SP, Brazil.; 2 Anaerobic Vaccines Section, Butantan Institute, São Paulo, SP, Brazil.; 3 Laboratory of Biochemistry and Biophysics, Butantan Institute, São Paulo, SP, Brazil.; 4 Biotechnology Center, Nuclear and Energy Research Institute (IPEN/CNEN/SP), São Paulo, SP, Brazil.; 5 Post Graduation in Health Sciences, Santo Amaro University, São Paulo, SP, Brazil.; 6 LIM49, Hospital das Clínicas HCFMUSP, School of Medicine, University of São Paulo, São Paulo, SP, Brazil.

**Keywords:** Radiation, Fragmentation, Enzymatic activity, Gamma rays

## Abstract

**Background:**

Tetanus toxin blocks the release of the inhibitory neurotransmitters in the central nervous system and causes tetanus and its main form of prevention is through vaccination. The vaccine is produced by inactivation of tetanus toxin with formaldehyde, which may cause side effects. An alternative way is the use of ionizing radiation for inactivation of the toxin and also to improve the potential immunogenic response and to reduce the post-vaccination side effects. Therefore, the aim of this study was to characterize the tetanus toxin structure after different doses of ionizing radiation of ^60^Co.

**Methods:**

Irradiated and native tetanus toxin was characterized by SDS PAGE in reducing and non-reducing conditions and MALD-TOF. Enzymatic activity was measured by FRET substrate. Also, antigenic properties were assessed by ELISA and Western Blot data.

**Results:**

Characterization analysis revealed gradual modification on the tetanus toxin structure according to doses increase. Also, fragmentation and possible aggregations of the protein fragments were observed in higher doses. In the analysis of peptide preservation by enzymatic digestion and mass spectrometry, there was a slight modification in the identification up to the dose of 4 kGy. At subsequent doses, peptide identification was minimal. The analysis of the enzymatic activity by fluorescence showed 35 % attenuation in the activity even at higher doses. In the antigenic evaluation, anti-tetanus toxin antibodies were detected against the irradiated toxins at the different doses, with a gradual decrease as the dose increased, but remaining at satisfactory levels.

**Conclusion:**

Ionizing radiation promoted structural changes in the tetanus toxin such as fragmentation and/or aggregation and attenuation of enzymatic activity as the dose increased, but antigenic recognition of the toxin remained at good levels indicating its possible use as an immunogen. However, studies of enzymatic activity of tetanus toxin irradiated with doses above 8 kGy should be further analyzed.

## Background

Tetanus is a highly lethal disease [1], its symptoms are trismus, spams, pain, muscular stiffness, dysphagia and autonomic dysfunction [2]. In underdeveloped countries, tetanus is still a public health issue and in 2015, caused about 57.000 deaths worldwide [3]. Tetanus is diagnosed by patient’s history and clinical signs [4], since there are no laboratory tests [5].

Tetanus is caused by tetanus toxin (TeNT) which, released by bacterial autolysis, infiltrates body fluids to nerve terminals [6]. TeNT enters the axonal retrograde transport pathway and reaches motor neurons located in the spinal cord [7]. Tetanus toxin inhibits synapses and blocks the release of inhibitory neurotransmitters glycine and GABA [8] by hydrolyzing the peptide bond between the synaptic vesicle protein VAMP (vesicle associated membrane protein) and synaptobrevine-2 [9]. Tetanus toxin is a 150 kDa polypeptide consisting of two chains: a 50 kDa light chain and a 100 kDa heavy chain linked by a single disulfide bond [10]. The light chain is a zinc-dependent protease that cleaves synaptobrevin and a heavy chain which is responsible for the internalization of tetanus toxin the neurons [11].

Currently, the best way to prevent tetanus is through vaccination [12]. Tetanus vaccine is produced by detoxification of tetanus toxin (TeNT) with formaldehyde [13] with the resulting toxoid being then adsorbed to an aluminum salt [14]. In some cases, these molecules contribute to adverse post-vaccine reaction [15]. Formaldehyde has been linked to some adverse events such as eczema [15] and contact allergies [16]. Aluminum compounds persist for up to 8-11 years after vaccination in the human body [17]. This fact, combined with repeated exposure, may be responsible for an over-activation of the immune system and subsequent chronic inflammation [17]. Contact allergy and small granulomas or nodules with persistent urticaria at the site can also occur [18].

Although the vaccine is effective, this detoxification process is being used since the early twentieth century [19] and remains like this until today [20]. However, new vaccinal strategies production have been developed, such as the use of ionizing radiation for detoxification of venoms and toxins as promising vaccine candidates [21], and also microorganisms [22].

Ionizing radiation promotes ionization and excitation of the medium [23], interacting with molecules [21,24]. Due to these properties, its use has contributed to considerable scientific advances [25] and to the development of vaccines, due to the search of the production improvement strategies for greater efficiency and safety [26]. In attenuated microorganisms vaccines, radiation proved to be an efficient technique for inactivating fungi (e.g. *Paracoccidioides brasiliensis)* [27] and parasites [28]. Also, radiation improved the immunogenicity against bacteria (e.g. *Streptococus pneumoniae)* [29] and irradiated viruses (e.g. Influenza A) [30] without the need of adjuvant. Previous studies with irradiated snake venoms have shown attenuation of toxicity when compared to non-irradiated ones [31] and greater immunogenic potential [32]. Ionizing radiation is a great tool for production of vaccine antigens, considering its effects in attenuating the toxicity, and also the production of better immunogens without the need of adjuvants and other chemicals, such as formaldehyde for detoxification [33,34].

Considering the importance of TeNT for the production and commercialization of vaccines and the promising use of ionizing radiation for the improvement of immunogens and the proposal of new vaccine candidates, since these irradiated molecules demonstrated an improvement in their immunogenic properties and a robust immune response without the use of adjuvants and chemical treatments for inactivation, the objective of this study was to evaluate the effect of^60^ Co gamma radiation on concentrated (unpurified) TeNT and its residual enzymatic activity following irradiation.

## Material and methods

### Experimental animals

To obtain antibodies against TeNT, C57Bl/6j (isogenic) mice (n = 5), weigh 20-22 g were used. These mice were obtained from the bioterium of the Medicine School of the University of Sao Paulo. These animals were kept in plastic cages with autoclaved pine shavings, with Nuvilab commercial feed and water *ad libitum*, their handling was in a accordance with the rules for the care of laboratory animals [35] and the “ Principles of ethics in animal experimentation (BCoAE Brazilian College of Animal Experimentation)” and the experimental protocols were approved by the Committee of Ethics and Research of the Institute of Tropical Medicine of Sao Paulo #000338A.

### Obtention of anti-TeNT serum

A group of five C57Bl/6j mice was immunized subcutaneously with three biweekly doses of 1.76 Lf (100 µL) of tetanus and diphtheria (TD) vaccine (Biological E. Limited). Fifteen days after the last dose, mice were euthanized and the whole blood was extracted by cardiac punction and placed in a single 1mL tube (Eppendorf). The whole blood samples were centrifuged at 3000 rpm for 5 minutes to separate the serum and stored at -20 ºC. TD vaccine was kindly provided by Dr. Marta Heloísa Lopes, Coordinator of the Immunization Center (HCFMUSP).

### Production of TeNT

Concentrated TeNT was kindly provided by the Bacteriology Service - Anaerobic Vaccines Section of the Butantan Institute, coordinated by Ms. Fernanda Lucio dos Santos Macarini. TeNT is obtained through cultivation of *Clostridium tetani* by continuous fermentation. After growth and bacterial lysis, the toxin is obtained by tangential filtration and concentrated by 30 kDa membranes [36].

### Protein quantification

Protein quantification was determined using a fluorimeter Qubit System (Thermo Fisher) using the reagents of the Qubit Protein Assay kit as recommended by the manufacturer.

### Irradiation of TeNT

Aliquots of TeNT (1.374 μg/mL) in aqueous solution were irradiated by cobalt-60 gamma radiation with doses ranging from 1 kGy to 8 kGy at a dose rate of 765 Gy/h using a GammaCell™ (Atomic Energy). The radiation was distributed homogeneously, without shielding and in the presence of oxygen. The entire process was carried out at room temperature and shortly after radiation, the samples were stored at 4 °C until use.

### Characterization of native and irradiated TeNT by polyacrylamide gel electrophoresis in the presence of SDS

Nine samples containing 5 μg of native TeNT (nTeNT) and irradiated TeNT 1 - 8 kGy (iTeNT) were added in 15 μL of reducing sample buffer 0.0625 M Tris (Synth)-HCl (VETEC), 2% SDS (Synth), 10% Glycerol (VETEC), 5% 2-Mercaptoethanol (Merck), 1M Urea, 5% Bromophenol Blue (Bio-Rad) or non-reducing buffer, with the same composition as above, excepted for the 2-Mercaptoethanol which was ommited, heated at 100 ºC for 5 minutes and applied to the gel. Six microliters of prestained protein standard (Bio-Rad) was loaded in each gel.

The electrophoretic mobility analysis (SDS PAGE), in a discontinuous and denaturant system was performed according to Laemmli [37] in Mini-Protean IV system (Bio-Rad). The stacking gel was prepared at a concentration of 4% and the resolving gel at a concentration of 7.5%, both are composed of acrylamide (Sigma Adrich)/bis-acrylamide (Merck). Electrophoretic migration was performed for approximately two hours (80 volts - 20-30 mA) in a running buffer solution [0.025 M Tris (Synth)-0.192M Glycine (Synth) pH 8.3]. The gels were stained with Coomassie Blue R250 [50% Methanol (Synth), 10% Acetic Acid (Synth), 0.1% Coomassie Blue R 250 (Bio-Rad)].

### Proteomic analysis

Proteomic analysis was carried out in collaboration with the Laboratory of Biochemistry and Biophysics of the Butantan Institute under the supervision of Dr. Daniel Carvalho Pimenta. All reagents used in the proteomic analysis (sections “Trypsin enzymatic digestion”, “Proteomic analysis of nTeNT and iTeNT peptides” and “MALDI-TOF Mass spectrometry of samples of nTeNT and iTeNT”) were purchased from Sigma Co. (St. Louis, MO, USA), unless otherwise stated.

*Trypsin enzymatic digestion*

The 100 kDa (heavy chain) and 50 kDa (light chain) gel bands of the nTeNT and iTeNT were selected, excised, and transferred to a 1.5-mL microtube. Then, the bands were destained with an ammonium bicarbonate solution (75 mM ammonium bicarbonate and 40% ethanol). After that, the supernatants were removed and incubated with 10 mM Ditiotreitol (DTT) at 37 ºC for 30 minutes (reduction step). Afterwards, 50 mM iodoacetoamide (IAA) was added and the samples were incubated at room temperature for 30 minutes in the absence of light. After incubation, the supernatants were removed and the gel bands were washed with 25 mM ammonium bicarbonate.

The samples were dehydrated by adding acetonitrile (ACN) (3 x 10 min.). Next, the samples were rehydrated with trypsin solution (10 ng/µL in 50 mM ammonium bicarbonate) for 45 minutes at 4 °C. Posteriorly, supernatants were removed and 50 mM ammonium bicarbonate was added and incubated for 18 hours at 30 °C.

To extract the peptides from the bands, a solution of ACN, 5% and trifluoroacetic acid (TFA) (1: 1), was added and the samples were placed in an ultrasound bath for 10 minutes. Finally, the supernatants were removed and placed in a new tube, and stored at − 80 °C before LC-MS analyses [38].

*Proteomic analysis of nTeNT and iTeNT peptides*

The trypsin digested Supernatants samples were analyzed by liquid chromatography-mass spectrometry (LC-MS), using an electrospray-ion trap-time of flight (ESI-IT-TOF) system coupled to a binary ultra-fast liquid chromatography system (UFLC) (20A Prominence, Shimadzu, Kyoto, Japan). The samples were resuspended in 0.1% acetic acid and loaded onto a C18 column (Discovery C18, 5 μm, 50 mm x 2.1 mm Sigma Co), with the solvents: (A) acetic acid/water (1:999, v/v) and (B): acetic acid/water/ACN (1:99:900, v/v/v). The column was eluted at a constant flow of 0.2 mL/min, the gradient varied from 5 to 40% of solvent B, over 35 minutes, at 40 °C and monitored at 214 nm by a Shimadzu SPD-M20A PDA detector. Mass spectrometry analysis was performed at source temperature of 200 °C. The interface voltage was set at 4.5 kV and the capillary voltage, at 1.8 kV. The fragmentation was induced by argon collision at 50% energy. The MS spectra were acquired under positive mode and collected in the range of 350 to 1400 m/z and the MS/MS spectra were collected in the range of 500 m/z to 1950 m/z. LCD Shimadzu raw data were converted (LCMS Protein Postrun, Shimadzu) to Mascot Generic Format (MGF) files prior to analyses. Peaks Studio V7.0 (BSI, Toronto, ON, Canada) was used for data processing [39]. Proteomic identification was performed according to the following parameters: error mass (MS and MS/MS) set to 0.1 Da; methionine oxidation and carbamidomethylation as variable and fixed modification, respectively; trypsin as enzyme; maximum missed cleavages (3), maximum variable post-translational modifications (PTMs) per peptide (3) and non-specific cleavage (one).

*MALDI-TOF mass spectrometry of samples of nTeNT and iTeNT*

Samples containing 40 µL (1.374 μg/mL) of nTeNT and iTeNT were lyophilized and resuspended with 5 µL of 0.1% TFA solution. One microliter of each sample was co-crystallized with 1 μL of sinapic acid matrix (saturated solution prepared in 50% ACN/ 0.1% TFA) directly on the metal sample plate. After drying at room temperature, they were analyzed using a matrix associated laser desorption ionization-time of flight MALDI-TOF/TOF (Axima Performance, Shimadzu®) mass spectrometer. The mass spectrum was obtained in the mass/charge 10,000 m/z to 200,000 m/z range, in linear positive mode.

### ELISA antigenicity analysis of nTeNT and iTeNT

A polystyrene plate with 96 high binding wells (Costar 3590) was sensitized with 100 µL/well (1 µg/mL) of nTeNT and iTeNT in 0.1M Sodium Carbonate buffer (Na_2_CO_3_-NaHCO_3_0.1 M, pH 9.5 - Sigma Co.) per well., for 18 hours in a humid chamber at 4 ºC. After that, the plate was washed five times with Phosfate Buffer Saline containing 0.02% Tween 20 (Synth) (PBS-T) using a HidroSpeed plate washer (Tecan). Blocking was done by incubation for one hour at 37°C with 250 μL of PBS-T containing 0.3% of skimmed milk powder (Molico) per well and washed five times with PBS-T. After blocking, 20 µL of serum from mice immunized with TD vaccine in hexaplicate were added in a dilution of 1/400 and incubated at 34 °C for one hour. Following incubation, the plate was washed five times with PBS-T. Afterwards, 20 µL of diluted anti-mouse IgG conjugate (1/10000) Peroxidase (Sigma Co.) was applied per well and incubated again at 34 ºC. The reaction was developed with 100 µL of OPD chromogenic solution (o-phenylenediamine 0.05%, Sigma Co. + citric acid 1% + Na2HPO4 1.45% in H2O, adding 10 μL 30% H_2_O_2_ for each 20 mL of the solution) and interrupted after 30 minutes with 4N HCl. Reading was performed on a Multi-mode Microplate Reader Spectrofluorimeter FilterMax F5 (Molecular Devices) at 492 nm [40]. Blank samples were used as controls, under the same conditions.

### Western blot reactivity analysis of nTeNT and iTeNT at different radiation doses

Protein separation was performed through SDS PAGE as described above. Then, the separated proteins were transferred to a 0.45-μm nitrocellulose membrane (Millipore) (10 volts, 40 minutes) in a Trans-Blot RD semi-dry transfer system (BIO-RAD), soaked in Towbin transfer buffer (25 mM Tris, 192 mM glycine, 20% methanol, pH 8.1-8.5 - Synth). The membranes were blocked with PBS-T containing 5% of skim milk for 1 hour under agitation. The membrane was incubated overnight with the mouse serum immunized with TD vaccine at a 1/400 dilution. Antigen-antibody binding was performed by incubation with mouse anti-IgG conjugated to peroxidase (Sigma Co.) for one hour. The reaction was then revealed with 3,3′-diaminobenzidine solution (DAB - Sigma Co.) (10 mL PBS, 10 mg DAB, 10 µL 30% H_2_O_2_) [41]. Between the steps, the membrane washes were performed with 0.05% PBS-T.

### Enzymatic activity assessment

The enzymatic activity assessment was performed according to the protocol described by Perpetuo et al. [42]. In a 96 well high binding black microplate (Costar), 200 µL (137 µg/mL) of nTeNT and iTeNT in Tris-NaCl pH 7.5 buffer (50 mM Tris, 150mM NaCl - Synth) were added (in duplicate) per well. Then, 5 mM FRET (Fluorescence Resonance Energy Transfer) substrate was applied to each well to start the reaction. Fluorescence values were measured every 30 seconds for 5 minutes. The fluorescence detector was adjusted to 320 nm excitation and 420 nm emission. Blank samples were used as controls, under the same conditions.

The FRET substrate was kindly provided by Dr. Ivo Lebrun from the Biochemistry and Biophysics laboratory at the Butantan Institute. The FRET substrate is composed by ortho-aminobenzoic acid (Abz) as fluorescent group and N-(2,4-dinitrophenyl) ethylenediamine (EDDnp) as quencher group. Abz is bound to the N-amino terminal of synaptobrevin (aminoacids residues 73-81) and EDDnp to the C-terminal carboxyl group: Abz-GASQ↓FETSA-Q-EDDnp. Arrow (↓) indicates the bond cleaved.

### Statistical analysis

Student's t-test was performed for the analysis of antigenicity and the calculation of linear regression for the analysis of residual enzymatic activity and were performed using the GraphPad Prism 6.0 statistical package.

## Results

### Characterization of the electrophoretic profile of nTeNT and iTeNT

**Figure 1.**
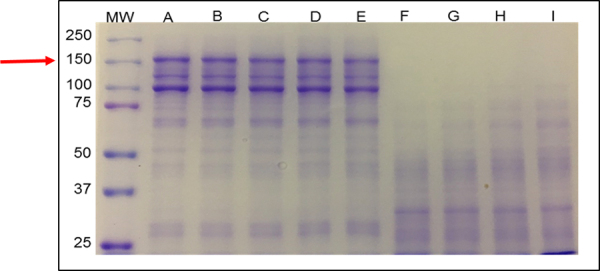
SDS-PAGE under non-reducing conditions (7.5% polyacrylamide gel, Mini Protean II System, Bio-Rad) of **(A)** native TeNT and TeNT irradiated with **(B)** 1 kGy, **(C)** 2 kGy, **(D)** 3 kGy, **(E)** 4 kGy, **(F)** 5 kGy, **(G)** 6 kGy, **(H)** 7 kGy and **(I)** 8 kGy. (MW) Molecular mass markers (Precision Plus Protein^TM^ Standards Bio Rad: mixture of 10 recombinant proteins, 10-250 kDa, 8 blue-stained bands and 2 pink reference bands - 25 and 75 kDa). Arrows point to tetanus toxin (150 kDa).

On non-reducing SDS-PAGE, we observed a heterogeneous profile, with a greater number of high molecular mass proteins and predominant bands at 100 kDa and 150 kDa of tetanus toxin (Figure 1). When comparing the fractions of the irradiated proteins with the proteins in their native state, we observed a slight change in the intensity of the bands up to the dose of 4 kGy. At doses of 5 kGy to 8 kGy, we noticed a smear and disappearance of the bands up to a mass of 100 kDa. In the range of 37 kDa up to 75 kDa, according to molecular mass markers, we noticed poorly defined band profiles, but the presence of low molecular mass bands below 37 kDa persisting up to the dose of 8 kGy. The formation of aggregates weighing over 150 kDa was not observed (Figure 1).

**Figure 2.**
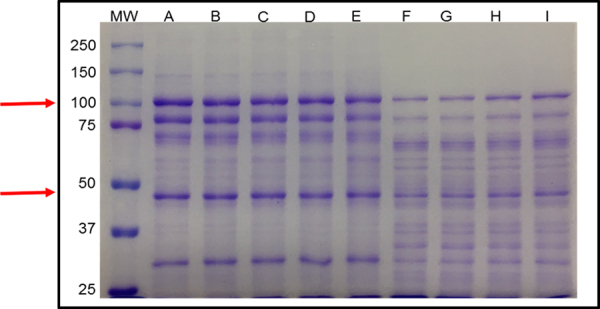
SDS-PAGE under reducing conditions (7.5% polyacrylamide gel, Mini Protean II System, Bio-Rad) of **(A)** native TeNT and TeNT irradiated with **(B)** 1 kGy, **(C)** 2 kGy, **(D)** 3 kGy, **(E)** 4 kGy; **(F)** 5 kGy; **(G)** 6 kGy, **(H)**7 kGy and **(I)**8 kGy. (MW) Molecular mass markers (Precision Plus Protein^TM^ Standards Bio Rad: mixture of 10 recombinant proteins, 10-250 kDa, 8 blue-stained bands and 2 pink reference bands - 25 and 75 kDa). Arrows point to tetanus toxin 100 kDa (heavy chain) and 50 kDa (light chain).

The electrophoretic profile obtained under reducing conditions revealed distinct patterns comparing to non-reducing conditions. In this profile, TeNT was distributed into a 100 kDa (heavy chain) and a 50 kDa band (light chain) (Figure 2). In the aliquots of proteins subjected to radiation, we noticed a gradual change in the profiles as the dose increased; however, the bands remain defined until the dose of 8 kGy. When comparing electrophoretic profile of native and irradiated TeNT, the dose of 1 kGy did not present significant changes. The electrophoretic profile of the tetanus toxin irradiated between the doses of 2 kGy and 4 kGy remained similar. From the dose of 5 kGy and higher, the bands are less expressive and concomitant with the appearance of bands of lower molecular mass which were not observed in TeNT irradiated with lower doses. Between the doses of 7 kGy and 8 kGy, there was an enlargement of the 100 kDa band. There is no evidence of formation of molecular mass aggregates above 100 kDa (Figure 2).

### Identification of TeNT peptides by gel digestion and mass spectrometry

**Figure 3.**
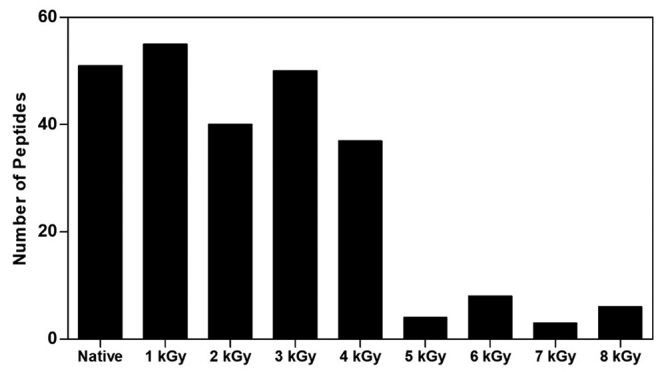
Determination of peptide number nTeNT and iTeNT with 1 kGy, 2 kGy, 3 kGy, 4 kGy, 5 kGy, 6 kGy, 7 kGy and 8 kGy by liquid chromatography-mass spectrometry (LC-MS), using an electrospray-ion trap-time of flight (ESI-IT-TOF) system coupled to a binary ultra-fast liquid chromatography system (UFLC) (20 A Prominence, ShimadzuKyoto, Japan).

TeNT was identified in all samples, but the number of identified TeNT´s peptides varied according to doses increasing. A pattern was observed in the number of TeNT´s peptides identified in the native sample, 1 kGy iTeNT and 3 kGy iTeNT, similarity was also observed in the 2 kGy iTeNT and 4 kGy iTeNT samples. From the 5 kGy iTeNTand doses above, the number of identified peptides decreased significantly without disparity up to the 8 kGy iTeNT (Figure 3).

### Structural analysis of nTeNT and iTeNT samples by mass spectrometry - MALDI-TOF

**Figure 4.**
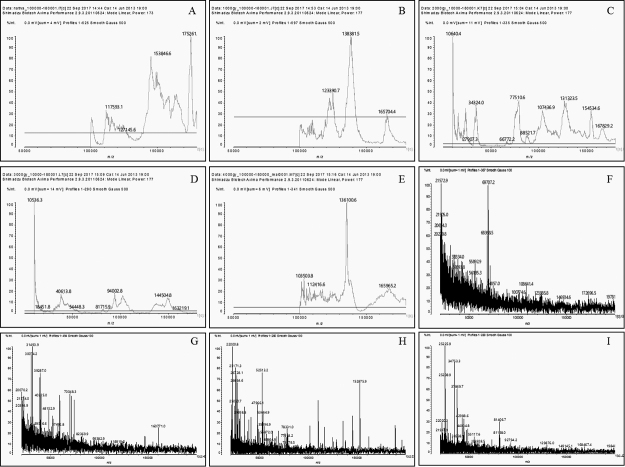
Mass spectra profiles of **(A)** native TeNT and TeNT irradiated with **(B)** 1 kGy, **(C)** 2 kGy, **(D)** 3 kGy, **(E)** 4 kGy, **(F)** 5 kGy, **(G)** 6 kGy, **(H)** 7 kGy, **(I)** 8 kGy by matrix associated laser desorption ionization-time of flight MALDI-TOF/TOF (Axima Performance, Shimadzu®) mass spectrometer.

In the nTeNT spectrum, two main charge/mass ratios were found: 175261 m/z and 153846.6 m/z (Figure 4A). In the spectra of irradiated proteins, the majority of the peaks mentioned were not observed. Smaller molecular mass fragments gradually formed up to the sample of iTeNT 4 kGy (Figure 4A, 4B, 4C, 4D and 4E). Most significant changes occurred from the iTeNT 5 kGy in which the presence of a greater number of peaks was observed. High mass peaks greater than 170,000 m/z were also noted from this dose (Figure 4F, 4G, 4H and 4I).

### Immunoreactivity characteristics of nTeNT and iTeNT

**Figure 5.**
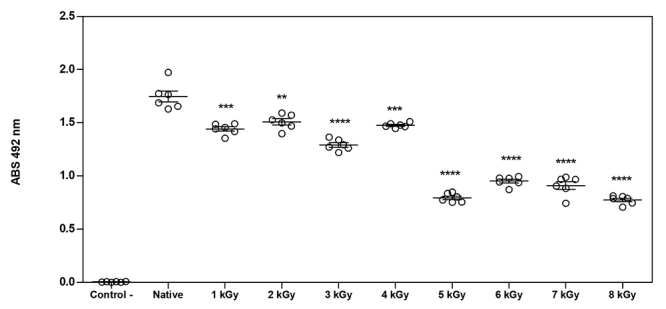
Antigenic characteristics of nTeNT and iTeNT was evaluated by ELISA using sera of immunized mice with Td vaccine. Results are presented as a binding of TeNT-specific-Abs to the same amount of nTeNT and iTeNTs. Samples are assessed in hexaplicates and results presented as mean ± SE. The statistical significance of the observed differences in binding of a given Abs to nTeNT and to iTeNT was calculated using Student’s t-test (*p < 0.05, **p < 0.005, ***p < 0.001).

Immunoreactivity analysis revealed that there was recognition of the IgG antibodies of C57Bl/6j mice immunized with the Td vaccine regardless of the dose that the toxin was submitted to. When comparing the immunoreactivity of the native toxin in relation to the irradiated toxins, we observed a significant difference in all doses. Among the irradiated samples, there was a gradual reduction in the immunoreactivity by antibodies. In antigens irradiated at 1 kGy, 2 kGy and 4 kGy, the significant difference was similar, followed by a greater loss of immunoreactivity in 3 kGy, and in samples from 5 kGy to 8 kGy (Figure 5).

**Figure 6.**
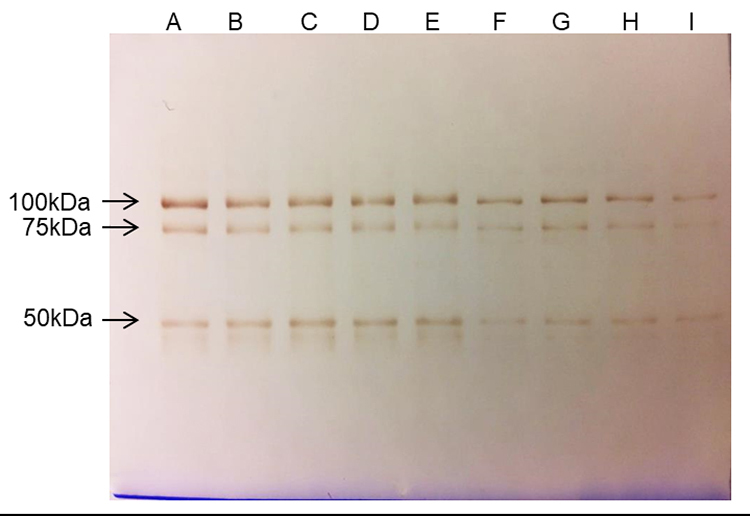
Western blot analysis of reactivity of TeNT-specific Abs toward H and L chain of TeNT. nTeNT and iTeNT was resolved on 7.5% polyacrylamide gel by SDS-PAGE under reducing conditions: **(A)** native; **(B)** 1 kGy; **(C)** 2 kGy; **(D)** 3 kGy; **(E)** 4 kGy; **(F)** 5 kGy; **(G)** 6 kGy; **(H)** 7 kGy; **(I)** 8 kGy.

Western blot analysis of the bands corresponding to the heavy chain (100 kDa) and the light chain (50 kDa) of the TeNT demonstrated that the two chains were recognized, showing that there are antigenic epitopes on the two polypeptides (Figure 6A). In the fractions of the irradiated samples, the antibodies were recognized in all profiles, as the dose increases, the recognition gradually decreases, with the 8 kGy band showing less reactivity (Figure 6).

### Enzymatic activity of native and iTeNT on FRET substrate

**Figure 7.**
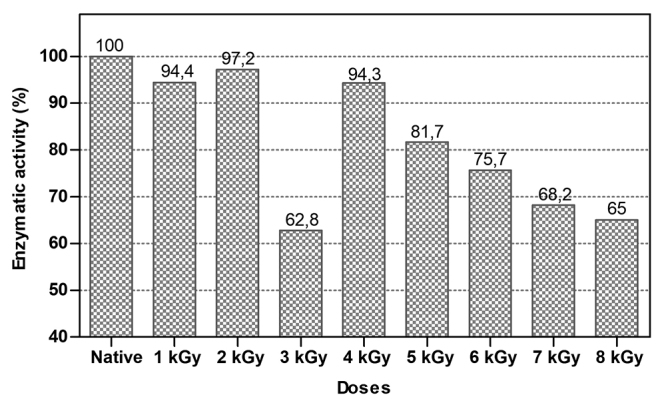
Enzymatic activity of nTeNT and iTeNT by FRET: Abz-GASQ↓FETSA-Q-EDDnp. Abz is bound to the N-amino terminal of synaptobrevin (aminoacids residues 73-81) and EDDnp to the C-terminal carboxyl group. Arrow (↓) indicates the bond cleaved.

Increasing the amount of radiation induced changes in the enzymatic activity of iTeNT. The samples of iTeNT1kGy, iTeNT2kGy and iTeNT4kGy showed similar loss of enzymatic activity, remaining at 94,4%, 97,2% and 94,3% respectively. From the iTeNT5kGy sample and higher, there was an attenuation of the enzymatic activity with the increase of the radiation dose: iTeNT5kGy - 81,7%, iTeNT6kGy - 75,7%, iTeNT7kGy - 68,2% and iTeNT8kGy - 65% of the activity. iTeNT3kGy showed greater loss of enzyme activity: 37,2%, probably, there was a contribution from environmental factors such as sample degradation (Figure 7). These data suggest that high doses of radiation can inactivate the TeNT.

## Discussion

Tetanus toxin is a potent neurotoxin that affects the release of neurotransmitters at nerve terminals and causes tetanus. Currently, the best tool to prevent tetanus is by immunization [43]. The process of vaccine manufacture involves cultivation of *C. tetani*, extraction, concentration the toxin supernatant, inactivation by formalin and purification. In our experiments we used the concentrated unpurified toxin obtained from the medium by filtration after the cultivation of *C. tetani* [44].

Tetanus vaccine has been used since 1924 and provides high protection. However, the vaccine manufactured with inactivated tetanus toxoid is related to effects caused after vaccination, toxicity, risks offered by the presence of vaccine components and by the usage of formaldehyde [45]. Currently, vaccines against tetanus that do not need adjuvants and the use of formaldehyde for their development are under investigation: as, for example, the use of fragment C linked to sulfhydryl [46], fragment C associated with flagellin [47] and recombinant vaccine of fragment C with the B subunit of the choleric toxin [48].

Initially, we carried out the characterization of concentrated and unpurified nTeNT and iTeNT. On non-reducing SDS-PAGE of nTeNT, we observed the 150 kDa band corresponding to the toxin and the presence of distinct proteins, with greater emphasis on the 100 kDa band. Tests carried out by Guilhen et al. [36] with the toxin sample under the same conditions as ours, obtained similar results and suggested three hypotheses for bands between 70 kDa and 60 kDa: (i) the light chain may have formed complexes with other proteins from the culture media or from the microorganism itself, (ii) these bands represent other proteins of *Clostridium tetani* unrelated to tetanus toxin and, (iii), degradation of the toxin heavy chain may have occurred. These hypotheses may also justify the 100 kDa band. Another event that might also have occurred is the disulfide bridge disruption and, thus, this band may correspond to the toxin heavy chain. A possible explanation for the presence of different *Clostridium tetani* proteins in the samples may be the result of bacterial lysis that is carried out to obtain the toxin right after the cultivation of the microorganism [49].

On reducing SDS-PAGE, the nTeNT is represented by the 100 kDa and 50 kDa bands, respectively. When we compare the profiles of tetanus toxin in reducing and non-reducing conditions, we observe different molecular masses. In the untreated sample with 2-mercaptoethanol, the toxin maintained its molecular mass of 150 kDa, whereas, when the sample was incubated with the reducing agent, there was a 100 kDa band and another 50 kDa band. Since 2-mercaptoethanol is a reducing agent and dissociates bonds made by disulfide bridges [50], the observed profile was expected, and the literature shows similar results obtained under the same conditions [51, 52].

The characterization of the irradiated samples demonstrated that the ionizing radiation caused a change in the molecular mass of the toxin as the radiation dose increased, and from the dose of 5 kGy and above this change was more representative. When considering the effects produced on proteins up to a dose of 5 kGy, previous studies without the use of the reducing agent present results similar to ours, however, in smaller doses. In the findings of Clissa et al. [53], the components with the highest molecular mass of *Crotalus durissus terrificus* venom were destroyed after being exposed to doses of 2, 3 and 5 kGy. A study carried out with crude venoms irradiated in solution of five species of the genera *Echis* and *Bitis* and six species of the genera *Naja* and *Dendroaspis*, aggregation was observed in doses higher than 4.5 kGy [54]. On the other hand, Caproni et al. [55] showed that radiation promoted structural changes characterized by higher molecular mass proteins, but did not show subunit dissociation even in the presence of a reducing agent, suggesting that radiation resulted in the formation of resistant intermolecular bonds to the agent. SDS PAGE revealed that intense fragmentation occurred after irradiation dose above 4 kGy and possibly aggregates. Our results are consistent with the results demonstrated by other authors, such as those found in a study using papain [56] and beta-galactoglobulin [57]. However, we still cannot prove that this change has occurred, since the authors report such observation in products irradiated with 10 kGy.

MALDI-TOF mass spectrometry confirmed the fragmentations observed in the gel caused by the use of radiation in all protein fractions exposed to different doses. In general, the literature shows observations similar to our MALDI-TOF analyzes. These fragmentations can be seen from the dose of 1 kGy. From the dose of 5 kGy, we observed the formation of peaks above 170000 m/z not observed in the spectrum of doses from 1 kGy to 4 kGy, which probably indicates the formation of aggregates and intense fragmentation. Studies carried out with crotamine have demonstrated an increase in mass at doses of 2 kGy and 10 kGy, which can be attributed to the oxidation of the venom [58], through this analysis, the formation of smaller fragments in the irradiated sample compared to native protein were also observed.

Ionizing radiation has been studied over the years for causing changes in the structure of proteins and for contributing to decrease the toxicity of molecules [59-61]. This conformational change and fragmentation occur through degradation and other actions caused by the direct and indirect action of ionizing radiation, where direct effects of radiation are caused when gamma rays and high-energy electrons interact directly with molecules. The greatest damage, on the other hand, may be indirectly, in which, from the radiolysis of water by ionization, reactive oxygen species (ROS) are formed that interact with amino acids through the addition or reduction of ions by oxidation [62]. After irradiation of molecules in solution, the ROS produced react very efficiently with proteins, favoring modifications such as dimerization and fragmentation [63]. After the generation of ROS, amino acids exposed to solvents are more likely to be oxidized by hydroxyl radicals [54]. Thus, the biological effects of ionizing radiation on proteins result in amino acid oxidation, oxidative cleavage of the protein skeleton and modification of the amino acid side chains [64].

At all doses analyzed, it was possible to observe that antigenic epitopes of TeNT remained with recognition by anti-TeNT IgG immunoglobulins. Despite the structural changes caused by the radiation process, we were able to observe the recognition of anti- TeNT antibodies against the toxins irradiated at different doses, with a gradual drop in proportion as the doses increase, but remaining with satisfactory levels until the highest dose. Antigenicity testing of TeNT treated with formaldehyde for its detoxification was also carried out by Metz et al. [14] and showed similar results. This may have been due to structural changes in specific epitopes on the toxin molecule. Similar results were also found in crotoxin [65] and total protein extract of *Toxoplasma gondii* [66] in which these antigens show conserved antigenic and immunological properties after the radiation process.

Enzyme activity test demonstrated attenuation of TeNT activity as the radiation dose increased, with the exception of the 3 kGy dose that had the lowest residual activity. Probably, the decrease in activity in this sample was influenced by environmental factors, such as the presence of proteases from the media which hydrolyzed the peptide bond [67], changes in pH that contribute to the unfolding [68] and the action of absorbed radiation [69]. The results obtained from this analysis are in accordance with previous studies that demonstrate the attenuation of the enzymatic activity by radiation as, for example, the loss of 52% of the enzymatic activity of the crude venom of the snake *Echis coloratus* irradiated at 3 kGy [70].

Although gamma radiation caused the same effects in these experiments, the radiation dose used is lower than ours, which suggests that TeNT presents some resistance to radiation. Such resistance may have been provided by some type of antioxidant molecule present in the culture media sample. The IB culture media produced at the Butantan Institute, in addition to other molecules, is composed of B vitamins [71], which are evidenced in the literature as potent antioxidants [72, 73]. Another fact that may have contributed to the resistance of the toxin to radiation is the presence of other proteins, providing mutual protection and competition for radicals formed by the indirect action of radiation. On the other hand, a study with enterotoxin of *Salmonella enterica* var *Typhimurium* demonstrated inactivation at the dose of 25 kGy, but a residual enterotoxicity at the dose of 10 kGy, which suggests that some proteins may need higher doses for the attenuation of their activity [74].

Extensive studies with irradiated protein have been performed throughout the last 30 years [75]. Importantly, these studies have shown considerable changes in the antigenic and immunogenic properties of irradiated proteins [76], which have been showing high immunogenicity of these vaccine candidates without the use of adjuvant [29, 30, 77].

## Conclusion

The data produced in this work showed that the irradiation of TeNT by cobalt-60 gamma rays, in different doses, altered the molecular structure as the dose increased, maintaining its antigenic capacity, but not showing satisfactory efficiency in the loss of enzymatic activity. Understanding the resistance mechanism of the TeNT to radiation, it is feasible to assure the safety of the molecule as a vaccine candidate. Although the TeNT remained with enzymatic activity above 50%, the data suggest the possibility of inactivation in higher doses and the use of ionizing radiation as an alternative method of detoxification of the TeNT for use as an immunogen. The use of ionizing radiation will also contribute to the improvement of the production process, optimizing the incubation time for detoxification, reduction of chemical residues resulting from the process and the possibility of making a vaccine without the need for adjuvant.

### Abbreviations

ACN: acetonitrile; DAB: 3,3′-diaminobenzidine solution; DTT: dithiothreitol; ESI-IT-TOF: electrospray-ion trap-time of flight; FRET: fluorescence resonance energy transfer; IAA: iodoacetoamide; iTeNT: irradiated TeNT; Lf: limit of flocculation; MALDI-TOF/TOF: matrix associated laser desorption ionization-time of flight; nTeNT: native tetanus toxin; OPD: orthophenyl diamine; PBS-T: phosfate buffer saline tween; TD: tetanus and diphtheria; TeNT: tetanus toxin; TFA: trifluoroacetic acid; UFLC: ultra-fast liquid chromatography system. 

## References

[B1] Stock I (2015). Tetanus and Clostridium tetani-a brief review. Med Monatsschr Pharm.

[B2] Vollman KE, Acquisto NM, Bodkin RP (2014). A case of tetanus infection in an adult with a protective tetanus
antibody level. Am J Emerg Med.

[B3] Kyu HH, Mumford JE, Stanaway JD, Barber RM, Hancock JR, Vos T (2017). Mortality from tetanus between 1990 and 2015: findings from the
global burden of disease study 2015. BMC Public Health.

[B4] Papadiochos I, Papadiochou S, Petsinis V, Goutzanis L, Atsali C, Papadogeorgaki N (2016). Trismus as a Clinical Manifestation of Tetanus: A Case
Report. J Oral Facial Pain Headache.

[B5] Bernardes M, Lo Presti S, Ratzan K (2018). A case of cephalic tetanus in an elderly patient with
trismus. Case Rep Infect Dis.

[B6] Ahaduzzaman M (2015). Updates on tetanus toxin: a fundamental approach. Vet Anim Res.

[B7] Lalli G, Bohnert S, Deinhardt K, Verastegui C, Schiavo G (2003). The journey of tetanus and botulinum neurotoxins in
neurons. Trends Microbiol.

[B8] Indrawattana N, Sookrung N, Kulkeaw K, Seesuay W, Kongngoen T, Chongsa-nguan M (2010). Human monoclonal ScFv that inhibits cellular entry and
metalloprotease activity of tetanus neurotoxin. Asian Pacific J Allergy Immunol.

[B9] Binz T, Rummel A (2009). Cell entry strategy of clostridial neurotoxins. J Neurochem.

[B10] Rossetto O, Scorzeto M, Megighian A, Montecucco C (2013). Tetanus neurotoxin. Toxicon.

[B11] Blum FC, Tepp WH, Johnson EA, Barbieri JT (2014). Multiple domains of tetanus toxin direct entry into primary
neurons. Traffic.

[B12] Borella-Venturini M, Frasson C, Paluan F, De Nuzzo D, Di Masi G, Giraldo M (2017). Tetanus vaccination, antibody persistence and decennial booster:
a serosurvey of university students and at-risk workers. Epidemiol Infect.

[B13] Thaysen-Andersen M, Jørgensen SB, Wilhelmsen ES, Petersen JW, Højrup P (2007). Investigation of the detoxification mechanism of
formaldehyde-treated tetanus toxin. Vaccine.

[B14] Metz B, Tilstra W, van der Put R, Spruit N, van den Ijssel J, Robert J (2013). Physicochemical and immunochemical assays for monitoring
consistent production of tetanus toxoid. Biologicals.

[B15] Aps LR de MM, Piantola MAF, Pereira SA, de Castro JT, Santos FA de O, Ferreira LC de S (2018). Adverse events of vaccines and the consequences of
non-vaccination: A critical review. Revista de Saude Publica.

[B16] Kuritzky LA, Pratt M (2015). Systemic allergic contact dermatitis after
formaldehyde-containing influenza vaccination. Journal of Cutaneous Medicine and Surgery.

[B17] Guimarães LE, Baker B, Perricone C, Shoenfeld Y (2015). Vaccines, adjuvants and autoimmunity. Pharmacological Research.

[B18] McNeil MM, DeStefano F (2018). Vaccine-associated hypersensitivity. Journal of Allergy and Clinical Immunology.

[B19] Ramon G (1924). Sur ia toxine et sur fanatoxine diphtériques. Ann Inst Pasteur.

[B20] Alsarraf H, Dedic E, Bjerrum MJ, Østergaard O, Kristensen MP, Petersen JW (2017). Biophysical comparison of diphtheria and tetanus toxins with the
formaldehyde-detoxified toxoids, the main components of diphtheria and
tetanus vaccines. Virulence.

[B21] Oliveira KC, Spencer PJ, Ferreira RS, Nascimento N (2015). New insights into the structural characteristics of irradiated
crotamine. J Venom Anim Toxins Incl Trop Dis.

[B22] Jwa MY, Jeong S, Ko EB, Kim AR, Kim HY, Kim SK (2018). Gamma-irradiation of Streptococcus pneumoniae for the use as an
immunogenic whole cell vaccine. J Microbiol.

[B23] Grosch DS, Hopwood LE (1979). Biological effects of radiations. Academic Press.

[B24] Azzam EI, Jay-Gerin J-P, Pain D (2012). Ionizing radiation-induced metabolic oxidative stress and
prolonged cell injury. Cancer Lett.

[B25] Cho K, Imaoka T, Klokov D, Paunesku T, Salomaa S, Birschwilks M (2019). Funding for radiation research: past, present and
future. International Journal of Radiation Biology.

[B26] Seo HS (2015). Application of radiation technology in vaccines
development. Clin Exp Vaccine Res.

[B27] do Nascimento Martins EM, de Andrade ASR, Kalkum M, Semis M (2017). Mouse immunization with radioattenuated yeast cells of
Paracoccidioides brasiliensis. Vaccines for Invasive Fungal Infections Methods in Molecular
Biology.

[B28] Oakley MS, Verma N, Zheng H, Anantharaman V, Takeda K, Gao Y (2016). Molecular markers of radiation induced attenuation in
intrahepatic Plasmodium falciparum parasites. PLoS One.

[B29] Babb R, Chen A, Hirst TR, Kara EE, McColl SR, Ogunniyi AD (2016). Intranasal vaccination with γ-irradiated Streptococcus pneumoniae
whole-cell vaccine provides serotype-independent protection mediated by
B-cells and innate IL-17 responses. Clin Sci.

[B30] David SC, Lau J, Singleton E V., Babb R, Davies J, Hirst TR (2017). The effect of gamma-irradiation conditions on the immunogenicity
of whole-inactivated Influenza A virus vaccine. Vaccine.

[B31] Souza FAD, Spencer PJ, Rogero JR, Nascimento N, Dal Pai-Silva M, Gallacci M (2002). 60Co gamma irradiation prevents Bothrops jararacussu venom
neurotoxicity and myotoxicity in isolated mouse neuromuscular
junction. Toxicon.

[B32] Rogero JR, Nascimento N (1995). Detoxification of snake venom using ionizing
radiation. J Venom Anim Toxins.

[B33] Ferreira RS, Nascimento N, Martinez JC, Alves JB, Meira DA, Barraviera B (2005). Immunization with native and cobalt 60-irradiated Crotalus
durissus terrificus venom in swiss mice: assessment of the neutralizing
potency of antisera. J Venom Anim Toxins Incl Trop Dis.

[B34] Pinho JR, Cardi BA, Andrade HF, Barr PJ, Bathurst IC, Vicente EJ (1995). Immunogenic properties of the M. leprae recombinant 18-kDa
antigen purified from Saccharomyces cerevisiae; enhancement of delayed-type
hypersensitivity after gamma-irradiation. Int J Lepr Other Mycobact Dis.

[B35] Clark J (1996). Guide for the care and use of laboratory animals. Institute of
Laboratory Animal Resources Comission on Life Sciences. National Research
Council.

[B36] Guilhen FB, Trezena AG, Prado SMA, Higashi HG, Sonobe MH (2014). Characterization of production processes for tetanus and
diphtheria anatoxins. Biologicals.

[B37] Laemmli UK (1970). Cleavage of structural proteins during the assembly of the head
of bacteriophage T4. Nature.

[B38] Westermeier R, Naven T (2002). Proteomics in Pratice.

[B39] Ma B., Zhang K., Hendrie C., Liang C., Li M., Doherty‐Kirby A., Lajoie G (2003). PEAKS: powerful software for peptide de novo sequencing by tandem
mass spectrometry. Rapid Commun Mass Spectrom.

[B40] Venkatesan P, Wakelin D (1993). ELISAs for parasitologists: or lies, damned lies and
ELISAs. Parasitol Today.

[B41] Towbin H, Gordon J (1984). Immunoblotting and dot immunobinding-current status and
outlook. J Immunol Methods.

[B42] Perpetuo EA, Juliano L, Juliano MA, Fratelli F, Prado SMA, Pimenta DC (2008). Enzymatic profiling of tetanus and botulinum neurotoxins based on
vesicle-associated-membrane protein derived fluorogenic
substrates. Protein Pept Lett.

[B43] Liang JL, Tiwari T, Moro P, Messonnier NE, Reingold A, Sawyer M (2018). Prevention of pertussis, tetanus, and diphtheria with vaccines in
the United States: Recommendations of the advisory committee on immunization
practices (ACIP). MMWR Recomm Reports.

[B44] World Health Organization (2017). Tetanus vaccines: WHO position paper - February
2017. RWkly Epidemiol Rec.

[B45] Yu R, Fang T, Liu S, Song X, Yu C, Li J (2016). Comparative immunogenicity of the tetanus toxoid and recombinant
tetanus vaccines in mice, rats, and cynomolgus monkeys. Toxins.

[B46] Yu R, Yi S, Yu C, Fang T, Liu S, Yu T (2011). A conformational change of C fragment of tetanus neurotoxin
reduces its ganglioside-binding activity but does not destroy its
immunogenicity. Clin Vaccine Immunol.

[B47] Lee SE, Nguyen CT, Kim SY, Thi TN, Rhee JH (2015). Tetanus toxin fragment C fused to flagellin makes a potent
mucosal vaccine. Clin Exp Vaccine Res.

[B48] Ibrahim EH, Asiri R, Al Syaad K (2018). Genetic fusion of tetanus toxin fragment C (Hc) gene to cholera
toxin subunit B (CTB) gene as a preparatory step for double vaccine
production. Gene Reports.

[B49] Chung Y-J, Jung M-Y, Lee J-A, Kim T-Y, Choe Y-K, Kim I-H (2016). Tetanus toxin production from Clostridium tetani, using a
casein-based medium in a single-use bioreactor. Biotechnol Bioprocess Eng.

[B50] Bodzon-Kulakowska A, Bierczynska-Krzysik A, Dylag T, Drabik A, Suder P, Noga M (2007). Methods for samples preparation in proteomic
research. J Chromatogr B.

[B51] Bayart C, Peronin S, Jean E, Paladino J, Talaga P, Le Borgne M (2017). The combined use of analytical tools for exploring tetanus toxin
and tetanus toxoid structures. J Chromatogr B Analyt Technol Biomed Life Sci.

[B52] Stojićević I, Dimitrijević L, Dovezenski N, Živković I, Petrušić V, Marinković E (2011). Tetanus toxoid purification: chromatographic procedures as an
alternative to ammonium-sulphate precipitation. J Chromatogr B Analyt Technol Biomed Life Sci.

[B53] Clissa PB, do Nascimento N, Rogero JR (1999). Toxicity and immunogenicity of Crotalus durissus terrificus venom
treated with different doses of gamma rays. Toxicon.

[B54] de la Rosa G, Olvera F, Cruz E, Paniagua D, Corzo G (2018). Use of irradiated elapid and viperid venoms for antivenom
production in small and large animals. Toxicon.

[B55] Caproni P, Baptista J, Almeida T de, Passos L, Nascimento N (2009). Study of irradiated bothropstoxin-1 with 60Co gamma rays: immune
system behavior. J Venom Anim Toxins Incl Trop Dis.

[B56] Varca GHC, Kadlubowski S, Wolszczak M, Lugão AB, Rosiak JM, Ulanski P (2016). Synthesis of papain nanoparticles by electron beam irradiation -
A pathway for controlled enzyme crosslinking. Int J Biol Macromol.

[B57] Oliveira CLP, Hoz L de la, Silva JC, Torriani IL, Netto FM (2007). Effects of gamma radiation on β-lactoglobulin: Oligomerization
and aggregation. Biopolymers.

[B58] Baptista JA, Vieira DP, Galisteo AJ, Higa OZ, Casare M, Yonamine CM (2009). Structure alteration and immunological properties of
60Co-gamma-rays irradiated bothropstoxin-I. J Radioanal Nucl Chem.

[B59] Le Maire M, Thauvette L, de Foresta B, Viel A, Beauregard G, Potier M (1990). Effects of ionizing radiations on proteins. Evidence of
non-random fragmentations and a caution in the use of the method for
determination of molecular mass. Biochem J.

[B60] Abdou F, Denshary E, Shaaban E, Mohamed M (2017). Assessment of the neutralizing potency of antisera raised against
native and γ-irradiated Naja nigricollis (black-necked spitting cobra) venom
in rabbits, concerning its cardiotoxic effect. Hum Exp Toxicol.

[B61] Calado T, Fernández-Cruz ML, Cabo Verde S, Venâncio A, Abrunhosa L (2018). Gamma irradiation effects on ochratoxin A: Degradation,
cytotoxicity and application in food. Food Chem.

[B62] Butler J, Land EJ, Swallow AJ (1984). Chemical mechanisms of the effects of high energy radiation on
biological systems. Radiat Phys Chem.

[B63] Li C, He L, Ma S, Wu W, Yang H, Sun X (2018). Effect of irradiation modification on conformation and gelation
properties of pork myofibrillar and sarcoplasmic protein. Food Hydrocoll.

[B64] Reisz JA, Bansal N, Qian J, Zhao W, Furdui CM (2014). Effects of ionizing radiation on biological molecules--mechanisms
of damage and emerging methods of detection. Antioxid Redox Signal.

[B65] Do Nascimento N, Seebart CS, Francis B, Rogero JR, Kaiser II (1996). Influence of ionizing radiation on crotoxin: biochemical and
immunological aspects. Toxicon.

[B66] da Costa A, Zorgi NE, do Nascimento N, Galisteo AJ, de Andrade HF (2018). Gamma irradiation of Toxoplasma gondii protein extract improve
immune response and protection in mice models. Biomed Pharmacother.

[B67] Jaenicke R (2000). Stability and stabilization of globular proteins in
solution. Journal of Biotechnology.

[B68] O’Brien EP, Brooks BR, Thirumalai D (2012). Effects of pH on proteins: Predictions for ensemble and
single-molecule pulling experiments. J Am Chem Soc.

[B69] Kempner ES (2001). Effects of high-energy electrons and gamma rays directly on
protein molecules. J Pharm Sci.

[B70] Samy EM, Shaaban EA, Kenawy SA, Abd Elfattah MA, Salama WH (2018). The impact of low doses of gamma radiation on Echis coloratus
venom and its fractions. Radiat Phys Chem.

[B71] Fratelli F, Siquini TJ, de Abreu ME, Higashi HG, Converti A, de Carvalho JCM (2009). Fed-batch production of tetanus toxin by Clostridium
tetani. Biotechnol Prog.

[B72] Dalto DB, Matte J-J (2017). Pyridoxine (Vitamin B₆) and the glutathione peroxidase system; a
link between one-carbon metabolism and antioxidation. Nutrients.

[B73] Ashoori M, Saedisomeolia A (2014). Riboflavin (vitamin B2) and oxidative stress: a
review. Br J Nutr.

[B74] Begum RH, Rahman H, Ahmed G (2011). Development and evaluation of gamma irradiated toxoid vaccine of
Salmonella enterica var Typhimurium. Vet Microbiol.

[B75] Murata Y, Nishikawa A, Nascimento N, HG H, Silva WD, Rogero JR (1990). Gamma irradiation reduces the toxic activities of Crotalus
durissus terrificus venom but does not affect their immunogenic
activities. Toxicon.

[B76] Spencer PJ, Do Nascimento N, Rogero JR (1997). Effects of Co 60 Gamma Radiation on the immunogenic and antigenic
proprietiers of Bothrops jararacussu Venom.

[B77] Shahrudin S, Chen C, David SC, Singleton E V, Davies J, Kirkwood CD (2018). Gamma-irradiated rotavirus: A possible whole virus inactivated
vaccine. PLoS One.

